# Sleep duration, sleep efficiency, and amyloid β among cognitively healthy later-life adults: a systematic review and meta-analysis

**DOI:** 10.1186/s12877-024-05010-4

**Published:** 2024-05-08

**Authors:** Chooza Moon, Aaron Schneider, Young-Eun Cho, Meina Zhang, Hellen Dang, Kelly Vu

**Affiliations:** 1 University of Iowa College of Nursing, 50 Newton Rd, Iowa City, IA 52242 USA; 2 University of Iowa College of Liberal Arts and Sciences Department of Health and Human Physiology, 225 S. Grand Ave., Iowa City, IA 52240 USA; 3 University of Iowa College of Pharmacy, 180 S. Grand Avenue, Iowa City, IA 52242 USA

**Keywords:** Amyloid plaque, Amyloid beta, Sleep, Sleep duration, Alzheimer’s disease

## Abstract

**Background:**

Abnormal amyloid β (Aβ) deposits in the brain are a hallmark of Alzheimer’s disease (AD). Insufficient sleep duration and poor sleep quality are risk factors for developing AD. Sleep may play a role in Aβ regulation, but the magnitude of the relationship between sleep and Aβ deposition remains unclear. This systematic review examines the relationship between sleep (i.e., duration and efficiency) with Aβ deposition in later-life adults.

**Methods:**

A search of PubMed, CINAHL, Embase, and PsycINFO generated 5,005 published articles. Fifteen studies met the inclusion criteria for qualitative syntheses; thirteen studies for quantitative syntheses related to sleep duration and Aβ; and nine studies for quantitative syntheses related to sleep efficiency and Aβ.

**Results:**

Mean ages of the samples ranged from 63 to 76 years. Studies measured Aβ using cerebrospinal fluid, serum, and positron emission tomography scans with two tracers: Carbone 11-labeled Pittsburgh compound B or fluorine 18-labeled. Sleep duration was measured subjectively using interviews or questionnaires, or objectively using polysomnography or actigraphy. Study analyses accounted for demographic and lifestyle factors. Based on 13 eligible articles, our synthesis demonstrated that the average association between sleep duration and Aβ was not statistically significant (Fisher’s Z = -0.055, 95% CI = -0.117 ~ 0.008). We found that longer self-report sleep duration is associated with lower Aβ (Fisher’s Z = -0.062, 95% CI = -0.119 ~ -0.005), whereas the objectively measured sleep duration was not associated with Aβ (Fisher’s Z = 0.002, 95% CI = -0.108 ~ 0.113). Based on 9 eligible articles for sleep efficiency, our synthesis also demonstrated that the average association between sleep efficiency and Aβ was not statistically significant (Fisher’s Z = 0.048, 95% CI = -0.066 ~ 0.161).

**Conclusion:**

The findings from this review suggest that shorter self-reported sleep duration is associated with higher Aβ levels. Given the heterogeneous nature of the sleep measures and outcomes, it is still difficult to determine the exact relationship between sleep and Aβ. Future studies with larger sample sizes should focus on comprehensive sleep characteristics and use longitudinal designs to better understand the relationship between sleep and AD.

**Supplementary Information:**

The online version contains supplementary material available at 10.1186/s12877-024-05010-4.

## Background

Alzheimer’s disease (AD) is a progressive neurodegenerative disease affecting one in ten adults over the age of 65 worldwide, which poses a considerable economic challenge [1]. More than 6.5 million older Americans suffered from AD in 2022, and the estimated cost for AD is $321 billion worldwide [2]. By 2050, the number of AD cases in the US is expected to reach 12.7 million individuals. Neurodegenerative processes associated with AD result in the accumulation of senile plaques and pathologic changes in Amyloid β (Aβ) throughout the brain, cerebrospinal fluid (CSF), and serum [3, 4]. AD biomarkers may be present even decades before clinical AD symptoms appear [5]. However, few effective disease-modifying treatments exist to delay the onset of AD symptoms. Thus, there is a pressing need to identify modifiable risk factors and develop novel interventions to decrease the risk of AD.

Alterations in sleep duration and efficiency can lead to numerous consequences for health and well-being and increase the risk of AD [6]. Current guidelines state that healthy sleep is a sleep duration of 7 or more hours per night for adults between 18 and 60, 7–9 h for adults between 61 and 64, and 7- 8 h for 65 years and older [7–9]. However, later-life adults who are older than 50 typically experience less than 7 h of sleep duration and 85% sleep efficiency compared to younger adults [10]. In particular, slow-wave sleep declines significantly with age [10]. In addition to the changes in sleep structures, sleep disorders including insomnia and sleep-disordered breathing increase with age [10]. Additionally, individuals with mild cognitive impairment or AD often experience disruptions in sleep and experience sundown syndrome. However, this condition often occurs years prior to impairment [11–13].

Sleep maintains brain and neural homeostasis [14]. During sleep, the brain controls Aβ peptide regulation [15], clears neurotoxins including Aβ plaques [16], and decreases systematic inflammation [17]. Thus, reductions in sleep duration or disruptions during sleep can influence the pathological changes of Aβ. Numerous recent papers and reviews focusing on the overall direction of sleep have suggested that sleep fragmentation or disruption is associated with AD via Aβ or tau pathology [15, 18–21]. These findings suggest that improving sleep efficiency and optimal sleep quantity could be an opportunity to prevent and delay AD pathology by decreasing Aβ deposition and tau hyperphosphorylation. Insomnia, sleep disordered breathing, and sleep fragmentation have been found to be associated with the risk of developing AD and related dementia [6]. However, researchers do not fully understand what the optimal sleep duration is to prevent AD.

Prior studies have speculated that shorter sleep duration can be associated with Aβ levels, because both total and partial sleep deprivation have been shown to increase Aβ levels in plasma [22, 23], CSF [24, 25], or brain [26–28]. For instance, Zhao et al. (2019) found that chronic sleep restriction was associated with increases in Aβ in a mouse model [28]. Kang et al. (2009) suggested that acute sleep deprivation can increase Aβ levels in animals via orexin regulation [29]. To better understand the magnitude of the relationship between sleep metrics and Aβ, a meta-analysis and/or systematic review is needed. However, few meta-analyses and systematic reviews have specifically focused on how sleep duration and/ or efficiency matters for Aβ accumulation in human studies on adults in later life. The purpose of this systematic review is to focus on the current state of science on how sleep duration/efficiency is associated with Aβ in the brain, CSF, and serum in older adults.

## Methods

The purpose of this study is to conduct a systematic review to evaluate how sleep duration and efficiency are associated with Aβ. The study was registered a priori with the International Prospective Register of Systematic Reviews (PROSPERO; registration no. CRD42021266789).

**Fig. 1:**
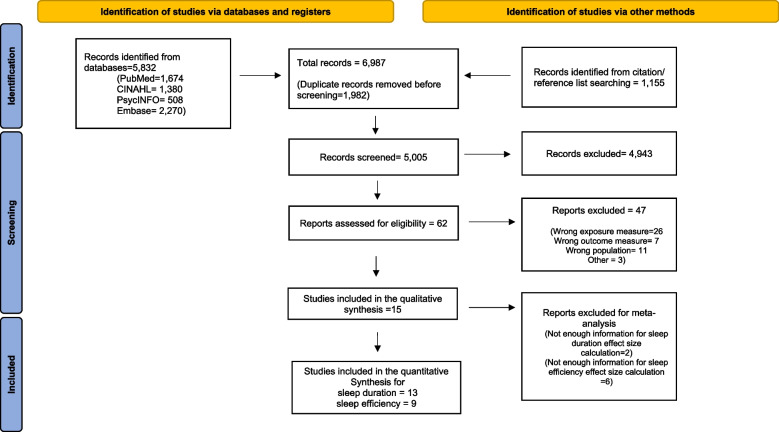
PRISMA 2020 flow diagram

This review was conducted following the guidelines of the Preferred Reporting Items for Systematic Reviews and Meta-Analyses (PRISMA) statement for reporting systematic reviews and meta-analyses [30]. Search strategies were developed with the assistance of a health sciences librarian with expertise in searching for systematic reviews. The flow diagram in Fig. 1 provides details on the search strategy and the number of articles each database yielded. Comprehensive strategies, including both index and keyword methods, were devised by the librarian and the primary author for the following databases: PubMed, CINAHL (EBSCO platform), Embase (Elsevier platform), and PsycINFO (EBSCO platform). To maximize sensitivity, no pre-established database filters other than the English language filter were used. The full PubMed search strategy, as detailed in Supplemental Table A, was also adapted for the other databases. In addition to the database searches, references and cited papers of the 1,156 relevant papers were located using the Scopus database.

Inclusion criteria for the qualitative synthesis were as follows: 1) observational studies with a longitudinal or cross-sectional design, 2) includes exposure variables of sleep duration and/ or sleep efficiency using subjective or objective measures, 3) has Amyloid β plaques (e.g., Aβ, Aβ_42_, Aβ_40_, Aβ_42_/Aβ_40_) as the outcome, 4) a human study of adults aged ^3^ 50 years old, and 5) recruited (or included) cognitively healthy individuals. An additional inclusion criterion for the quantitative synthesis was studies that reported sufficient data for examining the effect sizes, such as Pearson’s correlation (r), regression coefficient (β), means, standard deviations, t, F, or X^2^ values. We excluded studies 1) not written in English, 2) interventional studies, 3) non-peer reviewed papers, proceedings, editorials, and reviews, and 4) the study sample focused only on neurological conditions or sleep disorders. For the quantitative synthesis, we excluded studies that lacked or had inadequate inferential statistical results for calculating the effect size.

The initial search yielded 6,987 articles. After removing 1,982 duplicate articles, 5,005 articles were imported to the web-based systematic review application, Rayyan. The level of agreement between authors was determined using Cohen’s κ statistics. Four authors (CM, KV, HD, MZ) screened the abstracts and titles of the 5,005 articles based on the eligibility criteria (k = 0.49). Then, additional articles were removed leaving 62 full-text articles that were reviewed by four authors (CM, AS, YC, MZ) (k = 0.97). A total of 15 articles met the inclusion criteria for the qualitative synthesis and 13 studies met the criteria for quantitative synthesis of sleep duration, and 9 studies for sleep efficiency (Fig. 1 ). Disagreements were resolved through discussion among all authors until consensus was reached.

### Quality analysis

The risk bias of the selected papers was assessed independently by two reviewers using the National Institute of Health Study Quality Assessment Tool (2019) for Observational Cohort and Cross-Sectional Studies (https://www.nhlbi.nih.gov/health-topics/study-quality-assessment-tools). The internal validity of the studies were assessed based on 14 domains: 1) bias due to an unclear purpose, 2) bias due to an unclear specification of the population, 3) bias due to ineligible participants, 4) bias due to recruitment from a different population, 5) bias due to unclear power justification, 6) bias due to measure timing, 7) bias due to time frame, 8) bias due to outcome level, 9) bias due to invalid exposure measure, 10) bias due to frequency of the assessments, 11) bias due to the outcome measure, 12) bias due to an unblinded assessment, 13) bias due to loss during follow up, and 14) bias due to statistical analysis and confounding. For each domain, we categorized the risk of bias as either low or high risk. We rated an item “unclear risk” if there was no information about the risk of bias.

### Statistical analysis

We aggregated the effect sizes across the studies and calculated the publication bias, overall effect sizes, and Q statistics using Comprehensive Meta-Analysis (CMA) version 4 software (Biostat, Inc). We also calculated the effect sizes using Fisher’s Z as the effect size index after examining the available information on the correlation between sleep duration/sleep efficiency and Aβ using Fisher’s Z = 0.5*Log(1 + Corr)/(1-Corr) [31]. We used a Q statistic to evaluate the heterogeneity of variance. We also calculated the *I* ^2^ index using *I* ^2^ = 100% × (*Q* − degree of freedom)/*Q* to identify how the variance in observed effects reflected the variance in true effects rather than by random error. The random-effects model was applied in the current study because we expected that the sampling distribution varied across the studies and parameters were drawn from random variables [32–36]. We also ran subgroup analysis based on the sleep measure (i.e., self-report vs. objective). We used meta-regression analyses to investigate quantitative relationships between the dependent variable and covariate (i.e., sex). To consider the possibility of sampling bias from all possible samples, we assessed the studies for publication bias. First, we visually inspected the studies for symmetry of the funnel plot (Supplemental Figure A, B). Second, we ran Tweedie's Trim and Fill test to ensure that the publication bias could not reverse our estimate of the effect sizes [31].

## Results

Fifteen articles were included in the qualitative synthesis portion of this review. Thirteen studies were included for the quantitative synthesis of sleep duration and Aβ levels, and nine studies were included for quantitative synthesis of sleep efficiency and Aβ levels. Table 1 summarizes the study sample, design, and assessments of each study. Table 2 summarizes the study results and relevant information. Table 3 summarizes meta-analysis results.

**Table 1:** Study characteristics

First author (year)	Study Design	Sample name, country	Sample size / female n (%)	Mean age ± SD (years)	Exposure Assessment	Time window of sleep duration	Outcome Assessment	Covariates
Ju (2013) [37]	Cross-sectional	Washington University Knight Alzheimer's Disease Research Center & Adult Children Study, USA	142/ 84 (59.2%)	65.6 ± 8.2	Actigraphy (Actiwatch 2, Phillips Respironics)	2 weeks	CSF measured Aβ42	Age, sex, APOEε4 allele
Spira (2013) [38]	Cross-sectional	Baltimore Longitudinal Study of Aging, USA	70/ 33 (47%)	78.2 ± 7.9 when they completed PiB PET, and 76.4 ± 8.0 (range 53 – 91) when they completed sleep measures	Standardized interview of mean number of hours of sleep obtained each night during the prior month using the following response options: "more than 7"; "more than 6, up to 7"; "more than S, up to 6"; or "5 or fewer."	4 weeks	[C-11] PiB PET derived DVR	Age, sex, race, APOE ε4, depressive symptoms, BMI, cardiovascular or pulmonary disease, and use of sleep medication (any vs. none)
Spira (2014) [39]	Cross-sectional	From other studies or the community in Baltimore, MD, USA	13/6 (46%)	Normal = 69.4 ± 5.6 ;MCI = 75.2 ± 11.3	PSG	2 nights (The first night was for adaptation only second night data were used.)	18 F-florbetapir-PET Brain Imaging derived DVR	Age, sex, BMI
Sprecher (2015) [40]	Cross-sectional	Wisconsin Registry for AD (WRAP), USA	98/ 66 (67%)	Age at PiB PET scan = 62.4 ± 5.7; Age at sleep assessment 63.0 ± 5.6	Self -report Medical Outcomes Study Sleep Scale	4 weeks	[C-11] PiB PET derived DVR	Age, sex, APOE ε4, family history of Alzheimer's Disease, BMI
Brown (2016) [41]	Cross-sectional	Australian Imaging, Biomarkers and Life- style (AIBL) study of aging, Australia	184/108 (59%)	75.5 ± 6.1	PSQI	4 weeks	[C-11] PiB PET derived SUV, 18F-flutemetamol (FLUTE) derived SUVr, and 18F-florbetapir (FBP) derived SUVr	Age at PET scan, sex, years of education, depressive symptoms, time between sleep assessment and PET scan, Aβ burden, MMSE, BMI
Varga (2016) [42]	Cross-sectional	Community dwelling older adults from New York City Area, USA	36/19 (54%)	66.8 ± 8.2	PSG	1 night	CSF measured Aβ40 and Aβ42	Age, sex, APOE ε4, education, SWS duration, %TST in SWS, mean SWS bout length, total SWA, SWA in NREM cycles 1–4, cerebrospinal fluid biomarkers, medial prefrontal cortex volume
Hwang (2018) [43]	Cross-sectional	Brain Aging Study, Korea	133/71 (53%)	68.05 ± 7.68	Actigraphy (Actiwatch 2, Phillips Respironics, Murrysville, PA)	8 days	[C-11] PiB PET derived SUVr	Age, sex, depression symptoms, APOE ε4, selected actigraphic sleep and circadian variables
Gabelle (2019) [44]	Cross-sectional	MAPT-AAV45 sleep ancillary study, France	143/56 (39%)	median: 73 [70-85]	Standardized interview	4 weeks	18 F-florbetapir-PET Brain Imaging derived SUVr	APOE ε4, depression
Ettore (2019) [45]	Cross-sectional	INveStIGation of Alzheimer’s Predictors in Subjective Memory Complainers (INSIGHT)-preAD Study, Italy	68/48 (71%)	76.67 ± 3.52	Actigraphy (GT3X)	7 days	18 F-florbetapir-PET Brain Imaging derived SUVr	Age, sex, depression, MMSE
Lysen (2020) [46]	Cross-sectional	prospective Rotterdam Study cohort, Netherlands	Total sample: 4712/2700 (57%) Actigraphy sample: 849/433 (51%)	72 ± 8	PSQI/ Actigraphy	8 days	Plasma measured Aβ40 and Aβ42	Age, sex, education, presence of self-reported paid employment, time interval between measurements of sleep and biomarker, possible sleep apnea, batch number of biomarker analysis, habitual alcohol consumption, smoking status, BMI, hypertension, diabetes, T-cholesterol previous history of heart disease
Winer (2021) [47]	Cross-sectional	Anti-Amyloid Treatment in Asymptomatic Alzheimer Disease (A4) study, US, Canada, Australia, and Japan	4417/2618 (59%)	71.3 ± 4.8	Standardized interview question of "average total number of hours slept at night"	N/A	18 F-florbetapir-PET Brain Imaging derived DVR	Age, sex, years of education, self-identified race/ethnicity, number of APOE ε2 alleles, and number of APOE ε4 alleles
Liu (2021) [48]	Cross-sectional	Cognitive Disorders Clinics in the First People's Hospital of Foshan and communities, China	305/ 182 (60%)	69.07 ± 6.37	PSQI	4 weeks	Plasma measured Aβ40 and Aβ42	Model 1: Age, sex, education Model 2: APOE ε4, depressive symptoms, MMSE, BMI, exercise frequency, diabetes, hypertension, triglyceride, fasting blood glucose
Fu (2022) [49]	Cross-sectional	Chinese Alzheimer’s Biomarker and Lifestyle study, China	974/410 (42%)	61.6 ± 10.3	PSQI	4 weeks	CSF measured Aβ40 and Aβ42, phosphorylated tau (P-tau)	Age, sex, education, APOE ε4 status, hypertension, diabetes, coronary heart disease, stroke, smoking and drinking
Chu (2023) [50]	Cross-sectional	Community dwelling older adults from Shanghai Sixth People’s Hospital Affiliated to Shanghai Jiao Tong University School of Medicine	335/209 (62%)	64.4 ± 7.8	PSQI	4 weeks	18 F-florbetapir-PET Brain Imaging derived DVR and plasma measured Aβ40 and Aβ42	Age, sex, education, BMI, smoking, alcohol consumption, APOE ε4 status, Chinese version of Montreal Cognitive Assessment-Basic, hypertension, diabetes, hyperlipidemia, coronary artery disease, Aβ42/40, neurofilament light chain, sleep duration > 8 h, sleep disturbance
Blackman (2023) [51]	cross-sectional and longitudinal	European Prevention of Alzheimer’s Dementia Longitudinal Cohort Study, Europe	1168 (Subsample with longitudinal data = 332) /678 (58%)	64.7 ± 7.1	PSQI	4 weeks	CSF measured Aβ42	age, sex, research site and APOE-ɛ4 status (carriers versus non-carriers)

*Abbreviations: PiB* = Pittsburgh Compound B, *PET* = Positron emission tomography, *DVR* = Distribution volume ratio, *BMI* = body mass index, *CSF* = Cerebrospinal fluid, *PSG* = Polysomnography, *PSQI* = Pittsburgh Sleep Quality Index, *SUVr* = Standardized uptake value ratio, *MMSE* = Mini Mental State Examination, *TST* = Total sleep time, *SWS* = Slow wave sleep, *SWA* = Slow wave activity, *NREM* = Non-rapid eye movement

**Table 2:** Study results

First author(year)	Exposure categories	Outcome Definition	Results	Quantitative synthesis	
			Sleep Duration and Amyloid/ Sleep Efficiency and Amyloid	Sleep duration	Sleep efficiency
Ju (2013) [37]	Continuous	Aβ positive: Aβ 42 > 500 pg/ml	No significant association between actigraphy measured sleep duration and CSF Aβ42 levels; No differences in sleep duration between Aβ 42 > 500 pg/ml group and Aβ 42 ≤ 500 pg/ml group / Aβ 42 > 500 pg/ml had significantly higher sleep efficiency (83.7%) than those with Aβ 42 ≤ 500 pg/ml (80/4%)	Yes	Yes
Spira (2013) [38]	Continuous (“more than 7”; “more than 6, up to 7”; “more than 5, up to 6”; or “5 or fewer” were coded in 0 to 5)	Continuous	Shorter sleep duration was associated with greater Aβ levels, measured by mean cortical DVR (cDVR; B = 0.08, 95% confidence interval (CI) 0.03, 0.14, *p* = 0.005) and precuneus DVR (B = 0.11, 95% CI 0.03, 0.18, *p* = 0.007) /No report of the association between sleep efficiency and Aβ levels	Yes	No
Spira [39]	Continuous	Continuous	No significant association between sleep duration and Aβ levels / No report of association between sleep efficiency and Aβ levels	Yes	No
Sprecher (2015) [40]	Continuous	Continuous	No significant association between sleep duration and Aβ levels / No report of association between sleep efficiency and Aβ levels	Yes	No
Brown (2016) [41]	Continuous	Continuous	No significant association between sleep duration and brain Aβ burden. In addition, Sleep duration did not modulate the relationship between APOE e4 status and Aβ burden / No significant association between sleep duration and brain Aβ burden. In addition, Sleep efficiency did not modulate the relationship between APOE e4 status and Aβ burden	Yes	Yes
Varga (2016) [42]	Continuous	Continuous and Amyloid positive: CSF Aβ42 > 536.9 pg/mL)	No significant association between total sleep time and CSF Aβ42. No mean differences between Aβ positive and Aβ negative groups No significant association between total sleep efficiency and CSF Aβ42	Yes	Yes
Hwang (2018) [43]	Continuous	Aβ positive: SUVr > 1.21	No significant association between sleep duration and Aβ positivity No significant association between sleep efficiency and Aβ positivity	No	No
Gabelle (2019) [44]	Sleep duration (as a continuous variable, and categorized into < 6; 6–7; ≥ 7 h per night); sleep efficiency (less than 82.35%; 82.35%-93.75%; ≥ 93.75%)	Aβ positive: SUVr > 1.17 and SUVr > 1.22	No significant association between nighttime sleep duration (as a continuous variable or categorized into < 6; 6–7; ≥ 7 h per night) and Aβ positivity / No significant association sleep efficiency (as a continuous variable or categorized into < 82.35%; 82.35%-93.75%; ≥ 93.75%) and Aβ positivity	Yes	Yes
Ettore (2019) [45]	Continuous	Aβ positive: SUVr > 0.7918	No significant association between positive Aβ status and total sleep time / Significantly lower sleep efficiency (83.49%) in Aβ positive group (90.72%) than those with Aβ negative The sleep efficiency was associated with odds of having Aβ positivity (adjusted OR = 0.59, 95% CI = 0.44 ~ 0.72, *p* < 0.001)	Yes	Yes
Lysen (2020) [45]	Continuous	Continuous	No significant association between self-reported or actigraphically measured sleep duration and plasma Aβ levels No significant association between self-reported or actigraphically measured sleep efficiency and plasma Aβ levels	Yes	Yes
Winer (2021) [47]	Grouped by short sleep duration: less than or equal to 6 h, normal sleep duration: 7–8 h, and long sleep duration: more than or equal to 9 h	Continuous	Self-reported shorter sleep duration was linearly associated with higher Aβ levels (β [SE] = –0.01 [0.00]; *P* = .005). No difference in Aβ was found between long and normal sleep duration groups (β [SE] = 0.00 [0.01]; *P* = .99) / No report on the sleep efficiency	Yes	No
Liu (2021) [48]	Continuous Sleep duration (more than 7; 6–7; less than 6) Sleep efficiency (less than 65%; 65–74%; 75- 84%; ≥ 85%)	Continuous	Sleep duration was negatively associated with plasma Aβ42 level (β = − 0.267, 95% CI − 0.450 ~ − 0.084, *p* = 0.005) and Aβ42/Aβ40 ratio (β = − 0.058, 95% CI − 0.077 ~ − 0.039, *p* < 0.001) Sleeping less than 6 h was associated plasma Aβ42 level (β = 0.647, 95% CI 0.25 ~ 1.043, *p* = 0.0002) compared to sleeping longer than 7 h /Sleep efficiency was negatively associated with plasma Aβ42 level (β = − 0.025, 95% CI − 0.037 ~ − 0.013, *p* = 0.001) and Aβ42/Aβ40 ratio (β = − 0.004, 95% CI − 0.005 ~ − 0.002, *p* < 0.001) Having less than 65% of sleep efficiency is positively associated (β = 0.125, 95% CI 0.077 ~ 0.173, *p* < 0.001) with plasma Aβ42 level compared to sleep efficiency greater or equal to 85% Having sleep efficiency between 65% and 74 is positively associated (β = 0.0.059, 95% CI 0.016 ~ 0.102, *p* = 0.008) with plasma Aβ42 level compared to sleep efficiency greater or equal to 85%	Yes	Yes
Fu (2022) [49]	Continuous	Continuous	Sleep duration was significantly associated with plasma Aβ42 level (β = 2.71E-03; *P* = *p* < 0.01). *Nonlinear relationships /No report of the association between sleep efficiency and plasma Aβ42 level	No	No
Chu (2023) [50]	Sleep duration greater than 8 h (yes/no) for multivariate analysis and continuous for correlational analysis	Aβ positive based on visual rating and continuous for correlational analysis	Sleeping more than 8 h were associated with developing Aβ positive (OR = 4.167, *p* = 0.020) Sleep duration was not associated with Aβ42, Aβ40, or Aβ40/42 Sleep efficiency was not associated with Aβ42, Aβ40, or Aβ40/42	Yes	Yes
Blackman (2023) [51]	Sleep duration (categorized into < 5 h; 5-6 h; 6–7 h; ≥ 7 h per night); Sleep efficiency (categorized into < 65%;65–74%; 75–84%; ≥ 85%)	Aβ-positive: CSF Aβ42 < 1000 pg/ml	Cross-sectional analyses: Sleep efficiency was not associated with CSFAβ42 Longitudinal analyses: Sleep duration was not associated with CSFAβ42 Cross-sectional analyses: Sleep efficiency was not associated with CSF Aβ42 Longitudinal analyses: Sleep efficiency was not associated with CSF Aβ42	Yes	Yes

*Abbreviations: PiB* = Pittsburgh Compound B, *PET* = Positron emission tomography, *DVR* = Distribution volume ratio, *CSF* = Cerebrospinal fluid, *SUVr* = Standardized uptake value ratio

**Table 3:** Fisher’s Z score for association between sleep (duration and efficiency) with Amyloids beta

**Model**		**Effect size and 95% confidence interval**					**Test of null (2-Tail)**		**Prediction Interval**		**Between-study**		**Other heterogeneity statistics**			
Model	**Number Studies**	**Point estimate**	**Standard error**	**Variance**	**Lower limit**	**Upper limit**	**Z-value**	*P*-value	**Lower limit**	**Upper limit**	**Tau**	**TauSq**	**Q-value**	**df (Q)**	*P*-value	**I-squared**
**Sleep duration and Aβ**																
**Overall**	**13**	**-0.055**	**0.032**	**0.001**	**-0.117**	**0.008**	**-1.720**	**0.085**	**-0.247**	**0.138**	**0.082**	**0.007**	**44.44**	**12**	**0.000**	**72.99**
**Objective sleep**	**5**	**0.002**	**0.056**	**0.003**	**-0.108**	**0.113**	**0.038**	**0.969**	**-0.188**	**0.192**	**0.066**	**0.004**	**4.005**	**4**	**0.405**	**0.13**
**Subjective Sleep**	**9**	**-0.062**	**0.029**	**0.001**	**-0.119**	**-0.005**	**-2.146**	**0.032**	**-0.220**	**0.096**	**0.066**	**0.004**	**42.920**	**8**	**0.000**	**81.36**
**Sleep efficiency and Aβ**																
**Overall**	**9**	**0.048**	**0.058**	**0.003**	**-0.066**	**0.161**	**0.823**	**0.410**	**-0.342**	**0.437**	**0.154**	**0.024**	**66.532**	**8**	**0.000**	**87.98**
**Objective sleep**	**5**	**0.085**	**0.071**	**0.005**	**-0.054**	**0.225**	**1.199**	**0.230**	**-0.252**	**0.422**	**0.128**	**0.016**	**65.16**	**4**	**0.000**	**93.86**
**Subjective sleep**	**5**	**-0.007**	**0.061**	**0.004**	**-0.126**	**0.113**	**-0.107**	**0.915**	**-0.333**	**0.320**	**0.128**	**0.016**	**3.645**	**4**	**0.456**	**0.00**

### Qualitative synthesis

#### Study characteristics

The total number of participants of the 15 studies included in the qualitative synthesis was 11,295 individuals ranging from 13 to 4,712 participants. The overall demographics of this review are presented in Table 1. The countries included the United States (*n* = 5) [37–40, 42], Australia [41], France [44], Italy [45], Netherlands [46], South Korea [43], and China [48–50]. Winer and colleagues’ study collected data from participants in multiple countries, including the United States, Canada, Australia, and Japan [47]. Blackman et al. also involved 39 European organizations [51]. Most of the studies used a cross-sectional study design [37–50], and one study used a longitudinal design [51].

The mean age of the samples ranged from 61.6 to 75.7. Data from the studies included study subjects, and most studies had specific inclusion criteria for the mean age and cognitive status [37–51]. The rate of females included in the sample ranged from 42 to 71%. Except for the studies by Gabelle et al. and Fu et al., more females were included in the studies than male participants. All studies only included individuals who were cognitively healthy without any neurological or untreated psychological conditions or certain health conditions that may affect sleep and Aβ. Exclusion criteria for all studies in our analysis were low cognition or markers associated with cognitive impairment such as lesions, stroke, or neurological disorders [37–45, 47–51], other major illnesses [39, 42, 45, 48, 50], and drugs that are active in the CNS [39].

#### Quality assessment

**Fig. 2:**
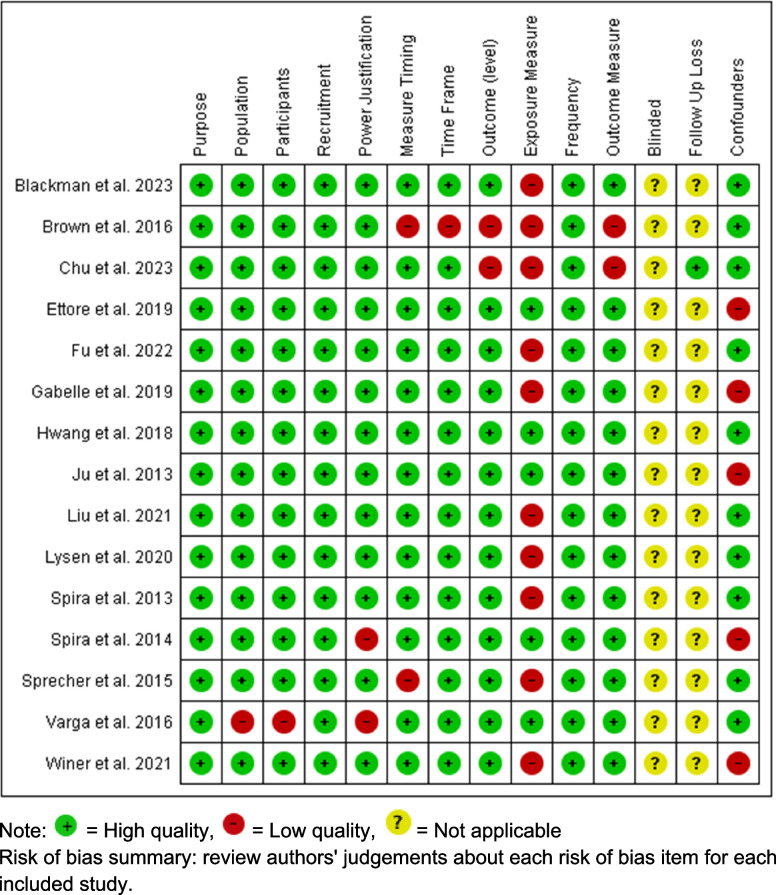
Risk of bias summary

Figure 2 illustrates the assessment of the risk of bias categories. Among the 15 studies, three had a moderate to high risk of bias due to measurement timing, the outcome measure, exposure measure, or the population and participants. Two of the 15 articles had a risk of bias related to a small sample size and population without any power justification. Two of the studies reported a high risk of bias related to the measurement timing, timeframe, and outcome and exposure variable. Ten of the 15 articles had a risk of bias related to the exposure measure using a self-report sleep question or questionnaire. Five articles had a risk of bias due to the limited number of confounding variables.

#### Sleep measures

Both subjective and objective sleep measures were used in the reviewed studies (Table 1). Five studies used objective measurements, including polysomnography (PSG) [39, 42] and actigraphy [37, 43, 45]. Overnight data were collected in studies with PSG. Of the three articles assessing sleep duration and sleep efficiency with actigraphy, two of the studies used Actiwatch 2 (Phillips Respironics) [37, 43]. Ettore et al. (2019) used a three-axis accelerometer (GT3X + , Actigraph Corp, Pensacola, FL). All actigraphy data were collected in 60-s epochs, and the duration of the actigraphy recording ranged from 6 to 14 days.

Ten of the reviewed studies used subjective measurements including the Pittsburgh Sleep Quality Index (*n* = 6) [41, 46, 48–51], a standardized interview to assess sleep duration (*n* = 3) [38, 44, 47], and the sleep scale in the Medical Outcomes Study (*n* = 1) [40]. The interviews included questions related to the duration of nighttime sleep, daytime sleep, total sleep time (daytime and nighttime sleep), and sleep efficiency [44]. Six articles used sleep duration as a categorical variable. Spira et al. coded the categorized sleep variable in a continuous manner by coding 0 for sleep duration longer than 7 h a night, and 3 for sleep duration of 5 h or less. The other two papers by Gabelle et al. and Winer et al. categorized sleep duration as shorter (6 h or less), normal (6—7 h and 6—8 h), and longer (≥ 7 h or ≥ 9 h) sleep duration. Chu et al. categorized sleep duration by the interquartile range (< 5 h, ≥ 5 h to < 6 h, ≥ 6 h to < 7 h, ≥ 7 h to < 8 h, > 8 h), and then used a dichotomous variable of sleep duration less than or equal to 8 and greater than 8 h. Blackman et al. also categorized sleep duration into < 5 h, 5-6 h, 6–7 h, and ≥ 7 h. The time window for the sleep duration assessment varied from 1 day to four weeks. Ten articles treated the sleep duration variable as a continuous variable. Ten of the reviewed studies reported sleep efficiency [37, 41–46, 48, 50, 51]. Of those articles, three studies categorized sleep efficiency [44, 48, 51] and the other seven studies treated sleep efficiency as a continuous variable.

#### Amyloid measures

The reviewed studies used a variety of Aβ measures (Table 1). Six studies measured Aβ concentration from bodily fluids including CSF (*n* = 4) [37, 42, 49, 51] and peripheral blood (*n* = 2) [46, 48]. CSF was obtained by lumbar puncture the morning after overnight fasting (from 8 to 10am) [37] or CSF samples collected between 11:00–13:00 [42]. In the two studies that tested peripheral blood, samples were collected in the morning after overnight fasting [46, 48], and were processed using plasma for further analysis. These studies measured Aβ_40_ and Aβ _42_ and assessed different combined ratios (e.g., Aβ _42,_/ Aβ_40_, P-tau/ Aβ _42_, T-tau/ Aβ _42,_ NFL/ Aβ _42_)[37, 42, 46, 48, 49] by an enzyme-linked immunosorbent assay (ELISA) [37, 42, 48–50] or by Simoa [46].

Nine studies measured Aβ using a PET scan [38–41, 43–45, 47, 50]. These studies obtained the amyloid deposit using two tracers: four studies used Carbone11 labeled Pittsburgh compound B (11C-PiB) [39–41, 43], six studies used fluorine 18 (18F) labeled tracers including 18F-florbetapir [38, 41, 44, 45, 47, 50], and one study used 18F-flutemetamol [41]. Brown et al. (2016) utilized data using three tracers: [C-11] PiB, and 18F-florbetapir, and 18F-flutemetamol [41].

Studies that conducted a quantitative assessment of Aβ used standardized uptake value ratios (SUVr) in four studies [41, 43–45], and the distribution volume ratio (DVR) in three studies [39, 40, 47]. Three studies with PET brain imaging used a cutoff to determine amyloid positivity. Ettore et al. (2019) used SUVr of 0.7918, Gabelle et al. (2019) used SUVr of 1.17, and Hwang et al. (2018) used SUVr of 1.21. Ju et al. (2013) used CSF Aβ_42_ of 500 pg/ml for the cut off.

Table 1 presents the covariate adjustments used in the statistical analyses of the studies. In general, the studies accounted for age and sex, except for Gabelle et al. (2019). Race and ethnicity were accounted for in two studies [38, 47]. Eleven studies controlled for APOEε4 allele [37, 38, 40, 42–44, 47–51]. Several studies also adjusted for education [41, 42, 46–50]. Clinical factors that were accounted for in the studies included depression [38, 41, 43–45, 48], and cognition status measured by the mini-mental state examination (MMSE) [41, 45, 48] or Montreal Cognitive Assessment (MOCA) [50]. Body mass index was a common lifestyle covariate considered in several studies [38–41, 46, 48, 50]. Other lifestyle covariates included hypertension [46, 48–50] and diabetes [46, 48–50]. Sleep and circadian rhythm variables were also included such as slow wave sleep [42], sleep disturbance [50], sleep apnea [46], or sleep medication [39]. Other factors included family history of AD, alcohol, and caffeine consumption [46, 49, 50], cholesterol levels [46, 48], and exercise [48].

#### Association between exposure and outcomes

Table 2 describes the findings of the reviewed studies for the qualitative synthesis. Five of the 15 articles [38, 47–50] found that shorter sleep duration was associated with higher Aβ. However, four of the studies reported the reverse association between sleep duration and PET-measured global and regional Aβ burden. Winer et al., (2021) found that self-reported shorter sleep duration was associated with greater 18 F-florbetapir-PET brain imaging derived DVR Aβ burden (β = –0.01; *p* = 0.005). Spira et al. (2014) also reported that shorter sleep duration was associated with greater Aβ burden, measured by mean cortical [C-11] PiB PET derived DVR (cDVR; β = 0.08, *p* = 0.005) and precuneus DVR (β = 0.11, *p* = 0.007). Longer total sleep time was associated with reduced 18 F-florbetapir-PET brain imaging derived SUVr global Aβ (β = -0.005; *p* = 0.03), reduced medial orbitofrontal Aβ (β = -0.009; *p* < 0.001), and reduced anterior cingulate Aβ (β = -0.011; *p* < 0.001). Sleep duration longer than 8 h was associated with having higher amyloid burden compared to sleep duration shorter or equal to 8 h (Odds Ratio = 4.167; *p* = 0.020) [50]. In addition to studies using PET, findings from a Chinese sample by Liu et al. (2021) also found that shorter sleep duration was associated with higher plasma Aβ_42_ (β = 0.495, *p* = 0.021) and Aβ_42_/Aβ_40_ ratio (β = 0.101, *p* < 0.001) [48]. Fu et al. (2021) also found a non-linear relationship indicating a decrease in CSF Aβ42 with shorter or longer sleep duration, with the extreme point being 6.23 h of sleep [49]. However, Blackman et al. (2023)’s cross-sectional analysis and longitudinal analysis did not identify any association between sleep duration and Aβ42 although the authors found significant associations between sleep characteristics and CSF P-tau and t-tau [51].

Of the reviewed studies, ten of the studies investigated sleep efficiency [15, 41–46, 48, 50, 51]. Three of the studies reported that sleep efficiency was associated with Aβ burden. Ettore et al. (2019) showed that lower sleep efficiency was found in the Aβ positive group, and increased sleep efficiency was associated with a 41% reduction of Aβ positivity (Odds Ratio = 0.59, < 0.001). Ju et al. (2013) reported that individuals with a low CSF Aβ_42_ level (≤ 500 pg/mL), which is indicative of amyloid deposition in the brain, had worse sleep efficiency than those with a normal CSF Aβ_42_ level (80.4% vs. 83.7%, *p* = 0.08), although sleep duration was not statistically significant. Liu et al. (2021) reported that sleep efficiency was negatively associated with the plasma Aβ42 level (β =  − 0.025, 95% CI − 0.037 ~  − 0.013, *p* = 0.001) and Aβ42/Aβ40 ratio (β =  − 0.004, 95% CI − 0.005 ~  − 0.002, *p* < 0.001). Specifically, experiencing less than 65% of sleep efficiency was positively associated (β = 0.125, 95% CI 0.077 ~ 0.173, *p* < 0.001) with plasma the Aβ42 level compared to sleep efficiency greater or equal to 85%. In particular, sleep efficiency between 65 and 74% was positively associated (β = 0.434, 95% CI 0.025 ~ 0.844, *p* = 0.038) with plasma Aβ42 level compared to sleep efficiency greater or equal to 85%.

#### Quantitative synthesis

**Fig. 3:**
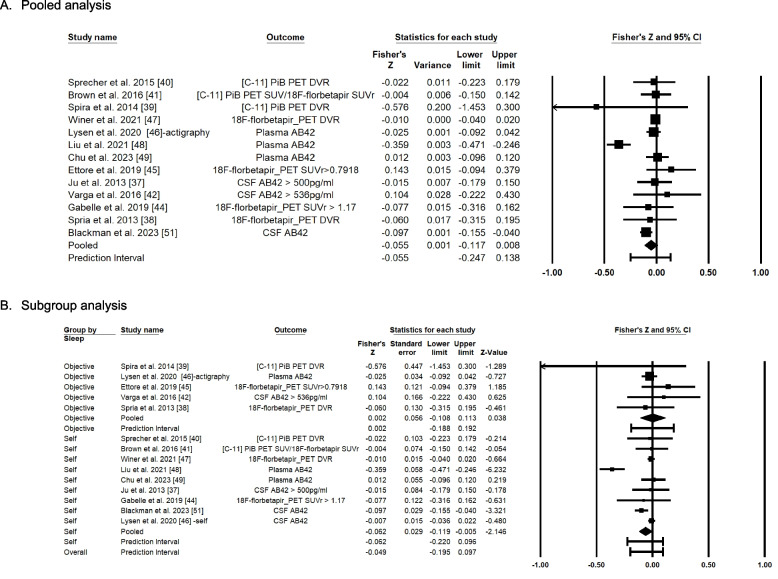
Forest plot of Fisher’s Z score for the association between sleep duration with Amyloid β

Thirteen eligible articles were used for the quantitative synthesis because the remaining studies were not included due to a lack of reporting information to calculate Fisher’s Z value. Because one study included both objective and subjective sleep data, we included objective sleep information to calculate the overall associations. However, we included the effect size information based on the self-report measures when we conducted the subgroup analysis. Figure 3 and Table 3 summarize the quantitative synthesis of the articles.

The findings demonstrate that the average association between sleep duration and Aβ was not statistically significant (Fisher’s Z = -0.055, 95% CI = -0.117 ~ 0.008) (Fig. 3A). The Z-value was -1.720 with *p* = 0.085. As shown in Table 3, for heterogeneity, our results indicate a Q-value of 44.44 with 12 degrees of freedom. The amount of between-study variance in the observed effect was less than we expected based on sampling error alone. The I^2^ statistic was 73%, indicating that 73% of the variance in observed effects reflect the variance in true effects rather than sampling error. Tau reflects the standard deviation of the true effect size, which is 0.007 in Fisher’s Z units.

For the subgroup analysis (Fig. 3B) based on the sleep measurement, we found that the average association between self-reported sleep duration and Aβ was significant. This finding indicates that longer self-reported sleep duration is associated with lower Aβ (Fisher’s Z = -0.062, 95% CI = -0.119 ~ -0.005) with a Z-value of -2.146 (*p*-value = 0.032) in nine studies. However, the average association between objectively measured sleep duration and Aβ was not statistically significant (Fisher’s Z = 0.002, 95% CI = -0.108 ~ 0.113) with a Z value of 0.038 (*p*-value = 0.969) in 5 studies. Furthermore, meta-regressions showed no impact of sex (coefficient = 0. 288; CI = -0.674 ~ 1.249) with Z value of 0.59 (*p*-value = 0.557). As shown in the standard error funnel plot by Fisher’s Z (Supplemental Figure A), the plot is slightly asymmetric, indicating that there could be minor publication bias from the included studies. This might be due to either our inability to identify studies with non-significant finings or failing to report non-significant findings [31]. Thus, we conducted an analysis using Tweedie’s Trim and Fill method for the overall relationship between sleep duration and Aβ, which demonstrated that even if we removed one study, the effect size remained statistically insignificant (Fisher’s Z = -0.054, 95% CI = -0.117 ~ 0.008). This finding may imply that some of the articles may not have presented the findings of non-significant results.

**Fig. 4:**
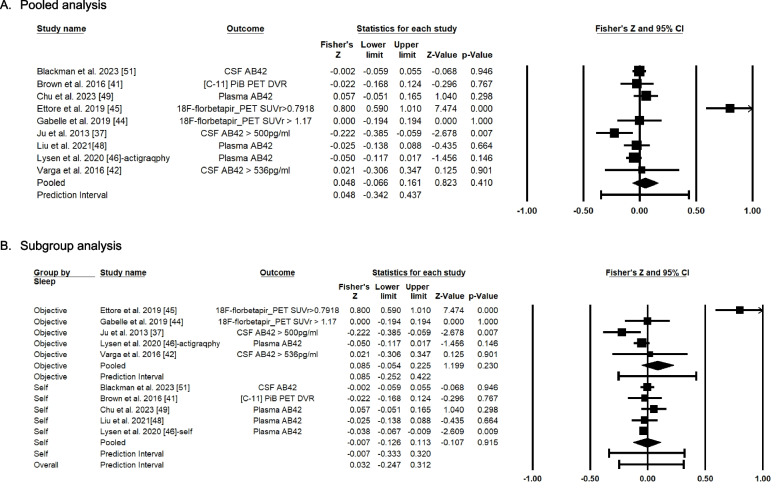
Forest plot of Fisher’s Z score for the association between sleep efficiency with Amyloid beta

For sleep efficiency, the findings from nine studies demonstrated that the average association between sleep efficiency and Aβ was not statistically significant (Fisher’s Z = 0.048, 95% CI = -0.066 ~ 0.161) (Fig. 4A). Figure 4 and Table 3 summarize the quantitative synthesis of the articles. The Z-value was 0.823 with *p* = 0.410. The Q-value is 66.532 with 8 degrees of freedom and *p* < 0.001. Using a criterion alpha of 0.100, we can reject the null hypothesis that the true effect size is the same in all of these studies. The I^2^ statistic was 88% and Tau-squared was 0.024 in Fisher's Z units, and Tau was 0.154 in Fisher's Z units. For the subgroup analysis based on the sleep measurement (Fig. 4B), we found that the average association between self-report sleep efficiency and Aβ was not significant (Fisher’s Z = -0.007, 95% CI = -0.126 ~ 0.113) with Z value of -0.107 (*p*-value = 0.915) in five studies. However, the average association between objectively measured sleep efficiency and Aβ was not statistically significant (Fisher’s Z = 0.085, 95% CI = -0.054 ~ 0.225) with a Z value of 1.199 (*p*-value = 0.230) in five studies. However, the meta-regression results indicated that higher proportion of female was associated with the higher correlation of sleep efficiency and Aβ (coefficient = 1.746, 95% CI = 0.345 ~ 3.136) with Z-value of 2.44 (*p*-value = 0.015). As shown in the standard error funnel plot by Fisher’s Z (Supplemental Figure B), the plot is slightly asymmetric, indicating that there could be minor publication bias from the included studies. This bias could be due to either our inability to identify studies with non-significant finings or failing to report non-significant findings [31]. We ran Tweedie’s Trim and Fill method for the overall relationship between sleep duration and Aβ, which demonstrated that even if we remove one study, the effect size remains statistically insignificant (Fisher’s Z = 0.048, 95% CI = -0.066 ~ 0.161). This finding may imply that some of the articles might not have presented the findings due to non-significant results.

## Discussion

This review synthesized fifteen studies for the qualitative synthesis and 13 studies for the quantitative synthesis focusing on sleep duration and Aβ and 9 studies for focusing on sleep efficiency and Aβ. This review adds to the current literature with an overview of the measurements and findings related to sleep and Aβ levels. Our meta-analysis findings indicate that there was a significant inverse relationship between self-report sleep duration and Aβ levels, indicating that self-report shorter sleep duration was associated with greater Aβ levels, with Fisher’s Z = -0.062 (95% CI = -0.119 ~ -0.005, *p* = 0.032). However, we did not find a significant overall relationship between sleep duration and Aβ levels. We also did not find a significant overall relationship between sleep efficiency and Aβ levels. Due to the heterogeneity among the published studies, no firm conclusions could be drawn.

We only found a significant inverse relationship between self-report sleep duration and Aβ levels. Prior research has demonstrated that chronic sleep restriction or deprivation of slow wave sleep can alter the diurnal fluctuation of CSF Aβ levels [24, 25, 52, 53]. Sleep deprivation may also impair human memory consolidation, in part by reducing the synthesis of proteins needed to support synaptic plasticity [14, 54–56]. In a meta-analysis by Wu and colleagues (2018), the authors suggested that there is a U-shaped relationship between sleep duration and cognitive disorders. Compared to the reference group (7 – 8 h per day), individuals with short or long duration had a higher risk of developing cognitive disorders, such as Alzheimer’s disease or dementia [57]. Both shorter sleep duration (< 7 h / night) and poor subjective sleep quality are important for cognitive function [58] and brain structures and functions [59, 60]. Furthermore, higher amyloid burden may be associated with worse sleep recollection in the self-report measures. However, more studies are needed using larger sample sizes, and a prospective design. Future studies on the magnitude of correlations of null results can shed light on the true relationship between sleep duration and Aβ.

The overall quantitative synthesis of sleep (duration and sleep efficiency) and Aβ revealed an effect size of -0.055 and 0.048, but they were not statistically significant, indicating that sleep may not be a primary factor in Aβ accumulation. Alternatively, these results may be due to moderators (i.e., APOE4, sex, age, family history, or unmeasured moderators), heterogenous outcome types of Aβ that may not provide consistent ideas, or publication bias due to insignificant results not being favorable for publication. There was considerable heterogeneity in the methods used in the reviewed studies to identify the relationship between sleep (duration and efficiency) and Aβ burden. It is intriguing that the results from the subgroup analysis differed when studying sleep duration and Aβ in studies using subjective and objective sleep measures. Future studies are needed to capture habitual sleep duration using an actigraphy with verification using a sleep diary for 7 to 14 days [61]. The use of a polysomnography would also provide insights on the structure of sleep and sleep disorders [62–64].

The majority of the studies used sleep duration as a continuous variable using multi-variate linear regression models. Winer et al. (2021), and Liu et al. (2021) used a categorial variable in the model and provided insights on the dose-dependent relationship between sleep duration and Aβ [47, 48]. Both studies indicated that a shorter sleep duration compared to the standard 7–8 h a night or 7 h or more sleep is associated with greater Aβ. Among the reviewed studies, only Fu and colleagues reported a non-linear relationship between sleep duration and CSF measured Aβ_42_ demonstrating lower Aβ_42_ values for shorter or longer sleep and the highest for 6.23 h [49]. Future studies using comprehensive and accurate assessments of sleep as well as a non-linear model would provide deeper insights on recommendations for sleep duration.

In addition to the considerable variability in sleep measurements, Aβ was also measured in different ways: CSF, serum sample, and PET to quantify the Aβ burden. Studies using PET used different tracers (e.g., 11C-PiB, 18F-florbetapir, 18F-flutemetamol) as well as different quantification methods. Most studies focused on global Aβ burden in the brain, but assessing both the overall levels of Aβ in PET as well as specific regional deposition could help us understand areas of the brain that may be affected more than other areas. This variability across the measurements prevented us drawing strong conclusions. However, it is promising in the current field of science to review data across different measures of Aβ accumulation. Although AD can be diagnosed at an autopsy [65], the US National Institute on Aging and Alzheimer’s Association has suggested using Aβ as well as tau and neurodegeneration to define and diagnose AD in both symptomatic and asymptomatic stages [66]. Increased accessibility to biomarkers and the potential for blood biomarkers or additional biomarkers in addition to Aβ would provide further information about the underlying disease progression in the future.

In addition to duration and efficiency of sleep, other sleep dimensions could be important factors for Aβ accumulation. The reviewed studies also identified a positive link between Aβ and different sleep characteristics including less adequate sleep, more sleep problems, and greater somnolence based on participants’ self-report perceptions [40], sleep quality [38], frequent napping [37], longer sleep latency [41, 45], greater sleep fragmentation [45], a higher apnea hypopnea index, and slow wave sleep time [39, 42]. These results may indicate that different dimensions of sleep could contribute more to Aβ burden than quantity of sleep.

The reviewed studies accounted for various demographic and clinical confounders in the multivariate models. Interestingly, we found that sex distribution could impact the relationship between sleep efficiency and Aβ although sex was not a significant moderator on the relationship between sleep duration and Aβ. Most of the reviewed studies accounted for age and sex, which are well-known confounders [10, 67–69]. Further stratified analysis or moderation analysis based on sex and age group will provide more accurate understanding of the covariates. Individuals with sleep disorders, underlying health or psychological conditions, medications, genetic factors, social determinants, high fat diets, and physical activity have different sleep quantity and quality [70–72]. These factors may also increase the amount of Aβ accumulation [73–80]. These confounding factors may play critical roles in determining the association between sleep duration and Aβ burden. However, these studies used a cross-sectional design, which prevents us from determining causal relationships. Specifically, some of the studies did not measure sleep and Aβ burden in a similar time period, so the results may not reflect a direct link between the two factors. Although researchers have speculated that the relationship between sleep and AD pathology could be bidirectional, there is limited evidence to support the longitudinal relationships [21, 81].

The strength of this review is that we examined current evidence related to sleep and Aβ. However, there are a few limitations. First, the study did not test for moderating effects of age, sex, or APOE4 status. Second, the current study only included publications written in English even though some important findings may have been published in different languages. Third, this review focused on sleep duration and efficiency, but other specific sleep characteristics could have more influence on Aβ pathology.

## Conclusions

The results of this systematic review suggest an inverse association between self-report sleep duration and Aβ. However, the relationships between sleep duration and Aβ accumulation as well as sleep efficiency and Aβ accumulation should be interpreted with caution. Researchers would greatly benefit from more studies using a longitudinal design, comprehensive sleep measure, a broad range of biomarkers, and larger sample sizes to advance scholarly understanding of the relationship between sleep and AD.

### Supplementary Information

**Supplementary Material 1.**

**Supplementary Material 2.**

## Data Availability

Data are available upon request to the corresponding author.

## Abbreviations

Aβ: Amyloid β
AD: Alzheimer’s disease
CSF: Cerebrospinal fluid
CMA: Comprehensive Meta-Analysis
DVR: Distribution volume ratio
MMSE: The mini-mental state examination
MOCA: Montreal Cognitive Assessment
PET: Positron emission tomography
PRISMA: Preferred Reporting Items for Systematic Reviews and Meta-Analyses
PSG: Polysomnography
SUVr: Standardized uptake value ratios
11C-PiB: Carbone11 labeled Pittsburgh compound B
18F: Fluorine 18
