# Supplementary figures and images for: Sleep duration, sleep efficiency, and amyloid β among cognitively healthy later-life adults: a systematic review and meta-analysis
Source: BMC Geriatr. 2024 May 8;24:408. doi: 10.1186/s12877-024-05010-4 (PMC11076214; doi:10.1186/s12877-024-05010-4)

Figure A. Sleep duration and Aβ funnel plot


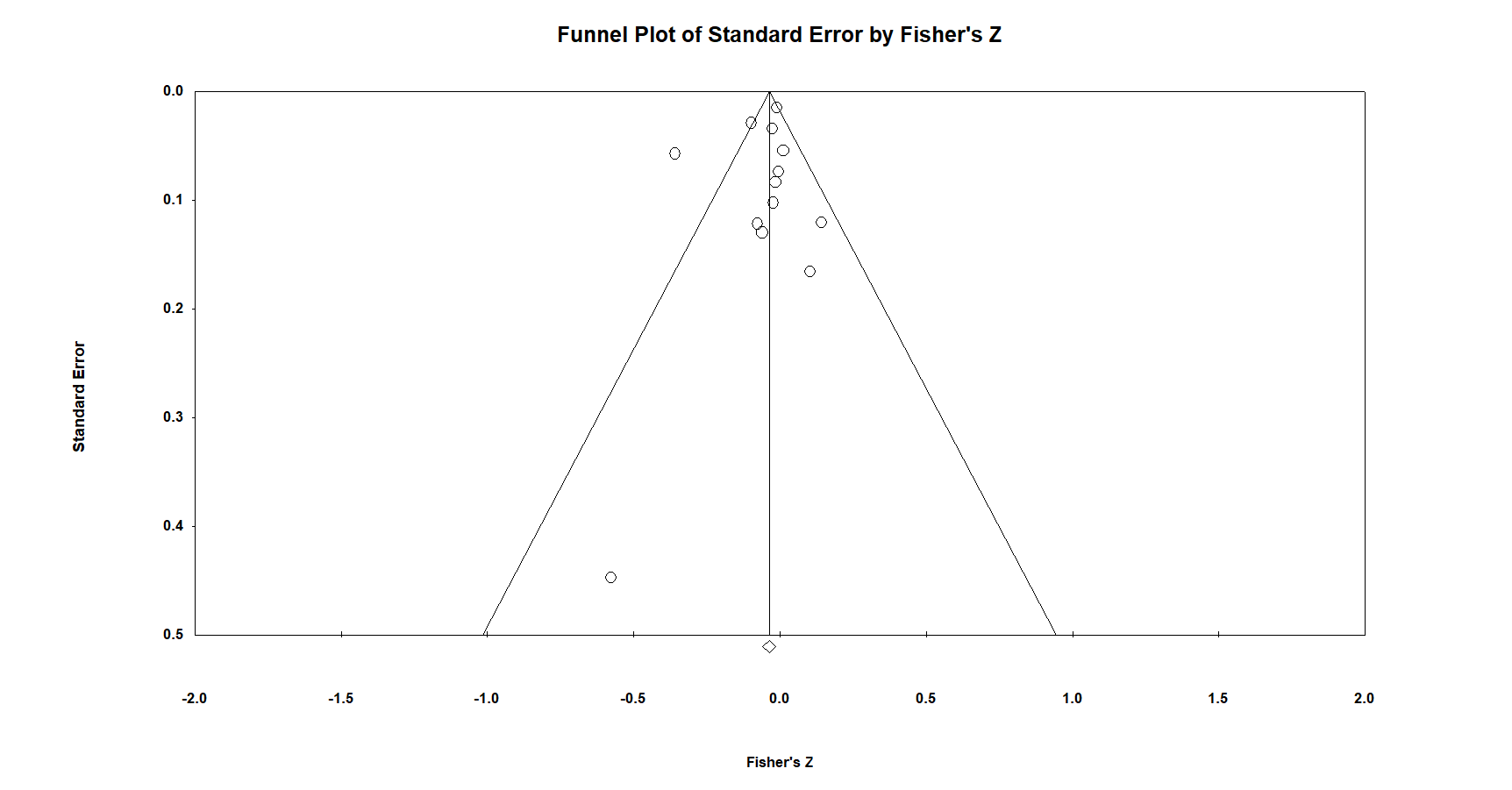


Figure B. Sleep efficiency and Aβ funnel plot


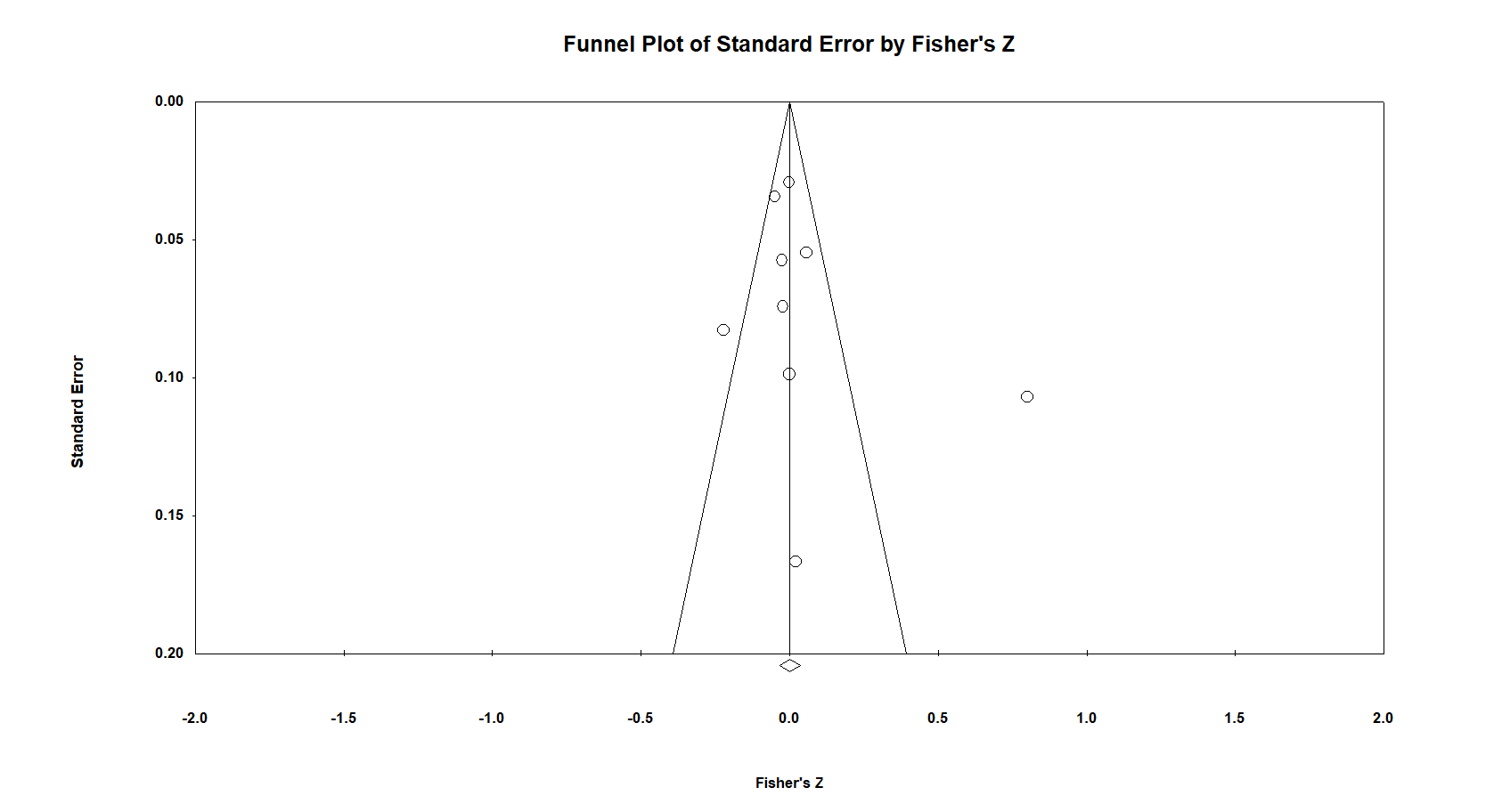

Supplement: Supplementary file 1 — Supplementary Material 1. [file 12877_2024_5010_MOESM1_ESM.docx]
