# Supplementary material for: Sleep duration, sleep efficiency, and amyloid β among cognitively healthy later-life adults: a systematic review and meta-analysis
Source: BMC Geriatr. 2024 May 8;24:408. doi: 10.1186/s12877-024-05010-4 (PMC11076214; doi:10.1186/s12877-024-05010-4)
Supplement: Supplementary file 2 — Supplementary Material 2. [file 12877_2024_5010_MOESM2_ESM.docx]

Table S1. Search strategies from the search engines

| Search Engine | Search Strategy | Search Date |
| --- | --- | --- |
|  |  | 2/27/2023 |
|  |  | N |
| **PubMed** | #1  "Amyloid beta-Peptides"[MeSH Terms] OR "tau Proteins"[MeSH Terms] OR "Presenilins"[MeSH Terms] OR amyloid [Title/Abstract] OR tau [Title/Abstract] OR neurofibrillary tangles [Title/Abstract] OR NFL [Title/Abstract] OR Neurofilament light chain [Title/Abstract] OR "Neurofilament Proteins"[Mesh]  #2  "Actigraphy"[Mesh] OR "Sleep"[Mesh Terms] OR "Sleep Phase Chronotherapy"[Mesh Terms] OR "Circadian Rhythm"[MeSH Terms] OR "Wakefulness"[MeSH Terms] OR "Chronobiology Disorders"[MeSH Terms] OR "Sleep Disorders, Circadian Rhythm"[MeSH] OR rest activity rhythm*[Title/Abstract] OR 24 hour activity [Title/Abstract] OR 24 h activity [Title/Abstract] OR circadian activity rhythm* [Title/Abstract] OR sleep wake pattern*[TItle/Abstract] OR bedtime [Title/Abstract] OR wake up time [Title/Abstract] OR Sedentary[Title/Abstract] OR sitting[Title/Abstract] OR reclining[Title/Abstract] OR screen time[Title/Abstract] OR LIPA[Title/Abstract] OR light intensity activity[Title/Abstract] OR light intensity physical activity[Title/Abstract] OR leisure time activity[Title/Abstract] OR MVPA [Title/Abstract] OR “moderate to vigorous physical activity” [Title/Abstract] OR high intensity activity [Title/Abstract] OR sleep [Title/Abstract] OR actigraphy [Title/Abstract] OR actimetry [Title/Abstract] OR periodicity [Title/Abstract] OR biorhythm* [Title/Abstract] OR biological rhythm [Title/Abstract] OR cyclicity [Title/Abstract] OR cyclicities [Title/Abstract] OR bioperiodicit* [Title/Abstract]  #3  "Editorial"[Publication Type] OR "Letter"[Publication Type] OR "Comment"[Publication Type] OR "Review"[Publication Type] OR "Case Reports"[Publication Type]  (#1 AND #2) NOT #3, with English language filter | 1674 |
| **Embase** | #1  'actimetry'/exp OR 'sleep'/exp OR 'chronobiology'/exp OR 'chronobiology' OR 'sleep disorder'/exp OR 'sleep disorder' OR 'wakefulness'/exp OR 'rest activity rhythm*':ab,ti OR '24 hour activity':ab,ti OR '24 h activity':ab,ti OR 'circadian activity rhythm*':ab,ti OR 'sleep wake pattern*':ab,ti OR bedtime:ab,ti OR 'wake up time':ab,ti OR sedentary:ab,ti OR sitting:ab,ti OR reclining:ab,ti OR 'screen time':ab,ti OR lipa:ab,ti OR 'light intensity activity':ab,ti OR 'light intensity physical activity':ab,ti OR 'leisure time activity':ab,ti OR mvpa:ab,ti OR sleep:ab,ti OR 'moderate to vigorous physical activity':ab,ti OR 'high intensity activity':ab,ti OR periodicity:ab,ti OR biorhythm*:ab,ti OR 'biological rhythm':ab,ti OR cyclicity:ab,ti OR cyclicities:ab,ti OR bioperiodicit*:ab,ti  #2  'amyloid beta protein'/exp OR 'presenilin'/exp OR 'presenilin' OR 'tau'/exp OR 'amyloid plaque'/exp OR 'amyloid plaque' OR 'neurofilament light chain' OR 'neurofilament protein'/exp OR amyloid:ab,ti OR tau:ab,ti OR 'neurofibrillary tangles':ab,ti OR nfl:ab,ti  #3  'chapter'/it OR 'conference abstract'/it OR 'conference paper'/it OR 'conference review'/it OR 'editorial'/it OR 'letter'/it OR 'review'/it  #1 AND #2 NOT #3, with English language filter | 2270 |
| **CINAHL** | #1  MH "Sleep+" OR MH "Wakefulness" OR MH "Chronobiology Disorders+" OR MH "Circadian Rhythm" OR MH "Sleep-Wake Transition Disorders+"  OR  TI (actigraphy OR actimetry OR  “rest activity rhythm*” OR “24 hour activity” OR “24 h activity” OR “circadian activity rhythm*” OR “circadian rhythm” OR  “sleep wake pattern*” OR bedtime OR “wake up time” OR Sedentary OR sitting OR reclining OR “screen time” OR LIPA OR “light intensity activity” OR “light intensity physical activity” OR “leisure time activity” OR MVPA OR “moderate to vigorous physical activity” OR “high intensity activity” OR sleep OR periodicity OR biorhythm* OR biological rhythm OR cyclicity OR cyclicities OR bioperiodicit* ) OR AB (actigraphy OR actimetry OR  “rest activity rhythm*” OR “24 hour activity” OR “24 h activity” OR “circadian activity rhythm*” OR “circadian rhythm” OR  “sleep wake pattern*” OR bedtime OR “wake up time” OR Sedentary OR sitting OR reclining OR “screen time” OR LIPA OR “light intensity activity” OR “light intensity physical activity” OR “leisure time activity” OR MVPA OR “moderate to vigorous physical activity” OR “high intensity activity” OR sleep OR periodicity OR biorhythm* OR biological rhythm OR cyclicity OR cyclicities OR bioperiodicit* )  #2  MH "Nerve Tissue Proteins+" OR MH "Proteins/ME" OR MH "Amyloids" OR MH "Neurons+/ME/PA" OR MH "Peptides+/ME" OR MH "Alzheimer's Disease/ME"  OR  TI ( "neurofibrillary tangle*" OR amyloid OR tau OR NFL OR “Neurofilament light chain” ) OR AB ( "neurofibrillary tangle*" OR amyloid OR tau OR NFL OR “Neurofilament light chain” )  #3  Publication Type: Book, Book Chapter, Book Review, Brief Item, Case Study, Editorial, Letter, Masters Thesis, Meta Analysis, Meta Synthesis, Questions and Answers, Review, Systematic Review  3/2/21 (#1 AND #2) NOT #3, limited to English and peer reviewed | 1380 |
| **PsycINFO** | #1  DE "Senile Plaques" OR DE "Beta Amyloid" OR DE "Neurofibrillary Tangles" OR DE "Tau Proteins"  OR  TI (amyloid OR tau OR “neurofibrillary tangles” OR NFL OR “Neurofilament light chain”) OR AB (amyloid OR tau OR “neurofibrillary tangles” OR NFL OR “Neurofilament light chain”)  #2  DE "Human Biological Rhythms" OR DE "Chronotype" OR DE "Cortisol Awakening Response" OR DE "CLOCK Gene"  OR DE "Sleep Wake Cycle" OR DE "Wakefulness" OR DE "Sleep Wake Cycle" OR DE "Actigraphy" OR DE "Sleep Quality" OR DE "Sleep" OR DE "Dreaming" OR DE "Napping" OR DE "NREM Sleep" OR DE "REM Sleep" OR DE "Sleep Onset" OR DE "Sleep Quality" OR DE "Snoring" OR DE "Sleep Wake Disorders" OR DE "Hypersomnia" OR DE "Insomnia" OR DE "Narcolepsy" OR DE "Parasomnias" OR DE "Sleep Apnea" OR DE "Sleepwalking"  OR  TI (actigraphy OR actimetry OR  “rest activity rhythm*” OR “24 hour activity” OR “24 h activity” OR “circadian activity rhythm*” OR “circadian rhythm” OR  “sleep wake pattern*” OR bedtime OR “wake up time” OR Sedentary OR sitting OR reclining OR “screen time” OR LIPA OR “light intensity activity” OR “light intensity physical activity” OR “leisure time activity” OR MVPA OR “moderate to vigorous physical activity” OR “high intensity activity” OR sleep OR periodicity OR biorhythm* OR biological rhythm OR cyclicity OR cyclicities OR bioperiodicit* ) OR AB (actigraphy OR actimetry OR  “rest activity rhythm*” OR “24 hour activity” OR “24 h activity” OR “circadian activity rhythm*” OR “circadian rhythm” OR  “sleep wake pattern*” OR bedtime OR “wake up time” OR Sedentary OR sitting OR reclining OR “screen time” OR LIPA OR “light intensity activity” OR “light intensity physical activity” OR “leisure time activity” OR MVPA OR “moderate to vigorous physical activity” OR “high intensity activity” OR sleep OR periodicity OR biorhythm* OR biological rhythm OR cyclicity OR cyclicities OR bioperiodicit*)  #3  #1 AND #2 with peer reviewed and English language filters | 508 |
